# Methadone for Emergence Delirium in Ambulatory Pediatric Strabismus Surgery

**DOI:** 10.31486/toj.23.0126

**Published:** 2024

**Authors:** Khaled A. Dajani, Bren Davis, Hussam Ghabra, Jakayla Harrell-Mohamed, Carol O. Carrillo, H. Sprague Eustis

**Affiliations:** ^1^ Department of Anesthesiology, Wolfson Children's Hospital, Jacksonville, FL; ^2^ Department of Anesthesiology, Nemours Children's Health, Jacksonville, FL; ^3^ Department of Ophthalmology, MedStar Georgetown University Hospital, Washington, DC; ^4^ Department of Anesthesia and Critical Care, King Abdulaziz University, Jeddah, Saudi Arabia; ^5^ Department of Anesthesiology, Touro Infirmary, LCMC Health, New Orleans, LA; ^6^ Department of Ophthalmology, Ochsner Clinic Foundation, New Orleans, LA

**Keywords:** *Anesthesia*, *delirium*, *methadone*, *pediatrics*, *strabismus*

## Abstract

**Background:** Emergence delirium in children following strabismus surgery is a distressing and potentially dangerous condition and is likely attributable to visual disturbances, pain, and anesthetic gases. We explored whether a single intraoperative dose of methadone could reduce emergence delirium.

**Methods:** Our study was an institutional review board–approved prospective, controlled, before-and-after investigation. Inclusion criteria were age <18 years and American Society of Anesthesiologists (ASA) classification 1 or 2. Patients were excluded for obesity, documented sleep apnea, significant neurologic disease, or inpatient status. Control group patients were recruited sequentially, and the anesthetic was performed per preference. The study group was recruited similarly and received an intravenous dose of methadone 0.15 mg/kg at induction. The primary outcome was peak score on the Pediatric Anesthesia Emergence Delirium (PAED) scale. Secondary outcomes included time to anesthetic emergence, postoperative pain scores, postanesthesia care unit (PACU) length of stay, and postdischarge respiratory complications.

**Results:** Forty-nine control group and 55 study group patients were recruited. No significant differences were found between groups for age, sex, weight, ASA classification, or duration of surgery. The control group received more preoperative midazolam, intraoperative fentanyl, and intraoperative ketorolac. Compared to the control group, the study group had 42% and 85% reductions in peak and severe PAED scale scores, respectively, in the PACU and required less rescue pain medications. Anesthetic emergence time and length of stay were not different between the groups. No significant postoperative complications occurred.

**Conclusion:** Emergence delirium following outpatient pediatric strabismus surgery was substantially mitigated by the use of intraoperative methadone without affecting PACU throughput. No significant complications occurred. Further study is warranted to corroborate routine use of this drug for emergence delirium.

## INTRODUCTION

Patient experience has become an important metric for health care systems seeking to maximize reimbursement, ensure patient loyalty, and most important, improve outcomes.^1^ Satisfaction of the parents is often used as a surrogate for patients in the pediatric sphere, and all validated experience surveys include questions about postoperative pain control, as well as overall nursing and medical care.^2^

Emergence delirium, also called emergence agitation, is at the very least a distressing phenomenon for parents to witness after their child's procedure and is highly likely to occur (40% to 86%) following strabismus surgery.^3-6^ Emergence delirium has a variable presentation that can include crying, aggression, and confusion during the early stages of anesthetic emergence and is potentially dangerous as it increases the risk of self-injury and staff injury.^7,8^ Pain is thought to be a major contributor to emergence delirium, as are preschool age and the use of modern low-solubility inhalational agents.^7,8^

Because of their increased half-life, single-dose long-acting opioids are superior to repeated interval dosing of shorter acting drugs in treating postoperative pain.^9-12^ Methadone is a μ-agonist with a rapid onset and an elimination half-life of 1 to 2 days, which has been demonstrated to reduce postoperative pain scores and opioid requirements for up to 30 days after outpatient discharge.^13^ Similar to ketamine, methadone also acts as an antagonist at the N-methyl-D-aspartate (NMDA) receptor, and ketamine in low doses has been shown to mitigate emergence delirium.^14,15^

Despite these theoretical considerations, to our knowledge, methadone has not been studied as an agent to reduce emergence delirium in pediatrics. The goal of this study was to determine the effectiveness of single-dose intraoperative methadone in reducing emergence delirium for children undergoing outpatient strabismus surgery. We also studied the effects this strategy had on anesthetic emergence, postanesthesia care unit (PACU) length of stay, postoperative pain control, and postdischarge respiratory complications.

## METHODS

Our study was a prospective, controlled, before-and-after investigation, approved by the institutional review board, with the requirement for written consent waived. The study was registered with ClinicalTrials.gov prior to the trial start or patient enrollment (NCT03778372). We recruited control group patients from January 2018 to September 2018, and recruited study group patients from January 2019 to August 2019. Patients undergoing strabismus surgery were included if they were <18 years of age and American Society of Anesthesiologists classification 1 or 2. Patients were excluded if they were scheduled for admission or if they were obese, had documented sleep apnea, or had significant neurologic disease. Participants were recruited sequentially with no randomization or blinding, and all anesthetics were performed by experienced, sub-boarded pediatric anesthesiologists. All unilateral or bilateral procedures were performed by an experienced sub-boarded pediatric ophthalmologist, involving 1 to 4 ocular muscles.

The decision to premedicate was at the discretion of the attending anesthesiologist. Patients received a standard intravenous (IV) or inhalational induction per age appropriateness. The airway was secured with an endotracheal tube or laryngeal mask airway. Control group anesthetics were carried out per preference. Study group patients received a single IV dose of methadone 0.15 mg/kg at induction based on actual body weight because obesity was an exclusion criterion. This dose was chosen after reviewing published dose-finding and clinical trial literature on methadone use perioperatively.^9-13^ No other preoperative or intraoperative narcotics were permitted for the interventional arm.

Nurses in the PACU used a standardized data collection sheet to document emergence delirium and pain. Patients who arrived asleep were noted to be so; otherwise, delirium was documented every 5 minutes for the first 30 minutes and then every 15 minutes until discharge using the Pediatric Anesthesia Emergence Delirium (PAED) scale.^16^ The PAED scale includes 5 criteria: (1) eye contact with the caregiver, (2) purposeful actions, (3) awareness of surroundings, (4) restlessness, and (5) consolability. Each of these 5 categories is given a score of 0 (not at all) to 4 (extremely). Pain was documented using either the Children and Infants Postoperative Pain Scale (CHIPPS) for patients <6 years or the visual analog scale (VAS) for patients 6 years and older.^17,18^ CHIPPS includes the criteria of crying, facial expression, trunk posture, lower limb posture, and restlessness, each scored between 0 (none) and 2 (most severe). The VAS consists of 6 faces, ranging from a green happy face to a red crying face, below a numeric scale from 0 (no pain) to 10 (worst possible pain). Vomiting and time of sustained consciousness were also documented. Given the long half-life of methadone, the pediatric anesthesiologist of record individually assessed study group patients with poorly controlled pain in the PACU. Parents were encouraged to contact anesthesiology with any nonurgent concerns after discharge and were educated about specific signs and symptoms that should prompt a call to emergency services. All patients were contacted by the pediatric ophthalmology clinic the day after surgery, and feedback was solicited regarding overnight vomiting, respiratory issues, and qualitative pain control.

Standard patient demographic, surgical, and anesthetic data were collected. The primary outcome was peak score on the PAED scale. Alpha was set at 0.05, and a power analysis was performed to calculate the sample size necessary to detect a 2-point difference, with power set at 80%. Secondary outcomes included anesthetic emergence time, time to sustained consciousness in the PACU, postoperative pain scores, PACU length of stay, and postdischarge respiratory complications. Statistical analysis was performed using JMP 12 (JMP Statistical Discovery LLC). Descriptive statistics are presented for demographic data, and a one-way analysis of variance or Fisher exact test was used to determine differences in outcomes. Multivariate regression analysis was performed on all statistically differing variables between groups to assess impact.

## RESULTS

Forty-nine control group and 55 study group patients were recruited for the study. Demographic and intraoperative data were similar between groups, although the control group received more oral midazolam for anxiolysis than the study group (Table 1). Both groups were also quite similar with respect to their surgical and anesthetic management, except the control group received more intraoperative fentanyl and ketorolac. The average intraoperative methadone dose for the study group was 3 ± 2.1 mg, while control group patients received fentanyl 23 ± 17 μg.

**Table 1.** Preoperative Demographics and Intraoperative Variables

Variable	Control Group, n=49	Methadone Group, n=55	*P* Value
Age, months	55 ± 48	62 ± 51	0.76
Sex			0.80
Male	22 (44.9)	26 (47.3)	
Female	27 (55.1)	29 (52.7)	
Weight, kg	21 ± 14	23 ± 16	0.66
American Society of Anesthesiologists classification	1.4 ± 0.6	1.6 ± 0.6	0.95
Preoperative midazolam, mg	10 ± 6	2 ± 1	**<0.0001**
Intraoperative ondansetron, mg	3 ± 1.4	3 ± 1.2	0.28
Intraoperative propofol, mg	61 ± 76	42 ± 59	0.10
Intraoperative fentanyl, μg	23 ± 17	0 ± 0	**<0.0001**
Intraoperative ketorolac, mg	13 ± 10	4 ± 8	**0.007**
Minimum intraoperative temperature, °C	35 ± 1	35 ± 1	0.61
Surgical duration, minutes	22 ± 6	22 ± 6	0.70

Notes: Data are reported as mean ± SD except for sex that is reported as n (%). Statistically significant differences are in bold.

Study group patients’ pain requirements differed significantly from those of the control group (Table 2). A greater percentage of control group patients received IV narcotic intervention, and their overall narcotic consumption was higher as well. Control group patients also received more nonnarcotic pain medicine, but no differences in vomiting or hypoxia were seen between the groups.

**Table 2.** Postoperative Recovery Data

Variable	Control Group, n=49	Methadone Group, n=55	*P* Value
PACU morphine equivalents, mg, mean ± SD	0.8 ± 2	0.1 ± 1	**0.02**
Intravenous narcotic intervention	6 (12.2)	1 (1.8)	**0.03**
Acetaminophen dose, mg, mean ± SD	144 ± 161	9 ± 49	**<0.0001**
Vomiting	1 (2.0)	3 (5.5)	0.37
Oxygen saturation <92%	0	0	–
Oxygen saturation, %, mean ± SD	96 ± 1.4	97 ± 2	0.67

Notes: Data are reported as n (%) unless otherwise indicated. Statistically significant differences are in bold.

PACU, postanesthesia care unit.

The primary outcome was PAED scores in the recovery room. Study group patients exhibited substantially less emergence delirium in the PACU compared to control group patients (Table 3). The severity of delirium was also mitigated in those receiving a single intraoperative dose of methadone. Multivariate regression analysis performed on statistically differing variables, including preoperative midazolam and intraoperative ketorolac, did not yield a correlation with the primary outcome. Despite a somewhat more protracted time to sustained consciousness for the study group patients, recovery room length of stay was similar between groups. Length of time asleep appeared directly related to resultant initial PAED scores, with longer sleep times correlating negatively with emergence delirium (Figure). Because of a communication oversight, pain scores were not collected for control group patients, but the mean maximum pain score for study group patients was 2 ± 3.

**Table 3.** Primary and Secondary Outcomes

Variable	Control Group, n=49	Methadone Group, n=55	*P* Value
Peak PAED score^a^	12 ± 5	7 ± 4	**<0.0001**
PAED score >10	28 (57.1)	15 (27.3)	**0.004**
PAED score ≥15	17 (34.7)	3 (5.5)	**0.001**
Emergence time, minutes	6 ± 4	7 ± 7	0.82
Emergence to consciousness, minutes	52 ± 42	68 ± 40	**0.04**
Maximum pain score^b^	NA	2 ± 3	–
PACU length of stay, minutes	89 ± 38	99 ± 34	0.08

^a^The Pediatric Anesthesia Emergence Delirium (PAED) scale includes 5 criteria scored from 0 to 4. Higher scores indicate worse outcomes.

^b^Because of a communication oversight, pain scores were not collected for control group patients. Pain for the study group patients was documented using either the Children and Infants Postoperative Pain Scale or the visual analog scale. Higher scores indicate worse outcomes.

Notes: Data are reported as mean ± SD except for PAED scores >10 and ≥15 that are reported as n (%). Statistically significant differences are in bold.

NA, not available; PACU, postanesthesia care unit; PAED, Pediatric Anesthesia Emergence Delirium scale.

**Figure.**
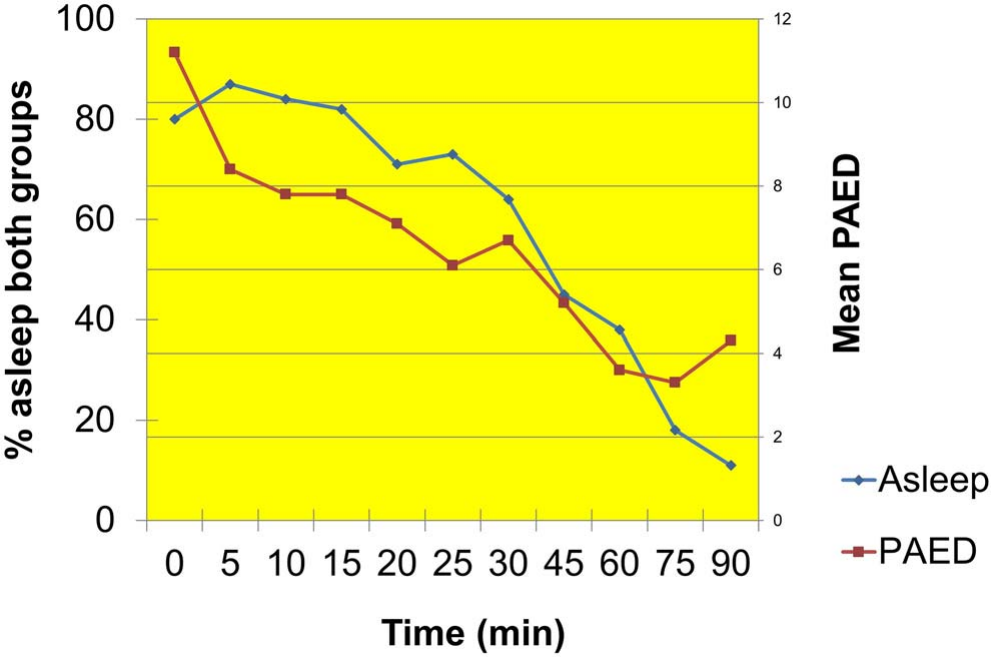
**Length of time asleep in the postanesthesia care unit after surgery correlated with steadily declining emergence delirium.** min, minutes; PAED, Pediatric Anesthesia Emergence Delirium scale.

One protocol violation was reported. A child in the 99th percentile for weight vs age received the weight-based methadone dose instead of being excluded. This patient did not experience any respiratory events but was extremely somnolent and had a prolonged recovery room stay prior to same-day discharge. No respiratory events on postoperative day 1 were reported by control group and study group parents who received a follow-up phone call at home per protocol.

## DISCUSSION

Although the United States is currently experiencing an adult opioid crisis, for children, the opposite situation has occurred, with the undertreatment of pediatric pain leading to international calls for transformative action.^19^ At our institution, “strabismus day” was a weekly struggle when the anesthesiologists and nurses did battle in the recovery room with postoperative emergence delirium and pain. In our study, a single intraoperative dose of methadone was demonstrated to reduce emergence delirium by 42% and mitigate the incidence of severe delirium (PAED score ≥15) by 85% without reducing throughput in the PACU.

Our selection of methadone as the study agent was founded on published observations. Emergence delirium is strongly related to pain, and methadone has been used for major inpatient surgeries such as scoliosis correction and for varied outpatient procedures to provide pain control.^9-13^ Emergence delirium seems to be related to young age and the ubiquitous use of modern-day inhalational agents, because otherwise moderate half-life opioids such as hydromorphone and morphine would be sufficient to eliminate emergence delirium.^7,8,20,21^

Sevoflurane and desflurane strongly precipitate emergence delirium through yet-unclear mechanisms, likely related to central nervous system (CNS) irritation caused by an imbalance between inhibitory and excitatory neurons.^7,8,20,21^ Glutamate, the primary excitatory transmitter in the CNS, binds NMDA receptors, which methadone antagonizes.^7,8,20,21^ Blockade of the NMDA receptor has shown much promise in the treatment of a wide variety of chronic pain syndromes related to neuronal excitation.^22^

In our study, more than half of the control group patients experienced emergence delirium in the recovery room, and one-third of patients had cases that were classified as severe. These results are consistent with existing literature and are in spite of the control group receiving more ketorolac and midazolam than the study group.^3-6^ A 2017 review of emergence delirium by Mason was unable to identify any role or benefit of benzodiazepines in mitigating emergence delirium,^21^ and our regression analysis agrees with Mason's conclusions.

At the outset of our study, several colleagues expressed concern over the potential for prolonged discharge times with the use of methadone, and even more important, the risk of respiratory complications. Although no statistically significant difference was seen in PACU length of stay between groups, this variable was not our primary outcome, and as such, our study was not powered to detect for this eventuality. The absence of a difference could reflect institutional practice.

No patients experienced respiratory complications postoperatively or on postoperative day 1 when our team called their parents at home to follow up. However, our single protocol violation illustrates the absolute necessity of careful patient selection and dosing when using methadone. The obese child experienced a PACU length of stay of nearly 180 minutes because of extreme somnolence.

Our study has several limitations. No control group pain scores were available for comparison to our study group, although some studies suggest that pediatric strabismus patients on average experience a pain score of 6 on several scales, while our study group's pain scores averaged 2 on the appropriate instrument.^23,24^ We used a controlled before-and-after design, a rigorous, well described approach that is less resource-intensive than randomized trials. Such studies carry several risks of bias; however, we followed best practices to minimize bias including the use of a control group, cohort matching, prospective data collection, and a written measurement tool.^25^

## CONCLUSION

Our conclusion is that the use of a single, intraoperative dose of methadone for outpatient pediatric strabismus surgery safely and substantially mitigates postoperative emergence delirium without reducing PACU throughput. This study was primarily an emergence delirium and safety study. Future directions may include a rigorous evaluation of postoperative pain control at home following discharge, as well as larger, randomized blinded trials.

## Notes

*This article meets the Accreditation Council for Graduate Medical Education and the American Board of Medical Specialties Maintenance of Certification competencies for Patient Care and Medical Knowledge.*
